# Clinical outcomes in children and adolescents initiating antiretroviral therapy in decentralized healthcare settings in Zimbabwe

**DOI:** 10.7448/IAS.20.1.21843

**Published:** 2017-09-01

**Authors:** Grace McHugh, Victoria Simms, Ethel Dauya, Tsitsi Bandason, Prosper Chonzi, Dafni Metaxa, Shungu Munyati, Kusum Nathoo, Hilda Mujuru, Katharina Kranzer, Rashida A. Ferrand

**Affiliations:** ^1^ Biomedical Research and Training Institute, Harare, Zimbabwe; ^2^ Department of Infectious Disease Epidemiology, London School of Hygiene and Tropical Medicine, London, UK; ^3^ Directorate of Health Services, Harare City Health, Harare, Zimbabwe; ^4^ Department of Paediatrics, University of Zimbabwe, Harare, Zimbabwe; ^5^ National and Supranational Reference Laboratory, Research Centre Borstel, Germany

**Keywords:** HIV, Africa, children, retention in care, outcomes

## Abstract

**Introduction**: Decentralized HIV care for adults does not appear to compromise clinical outcomes. HIV care for children poses additional clinical and social complexities. We conducted a prospective cohort study to investigate clinical outcomes in children aged 6–15 years who registered for HIV care at seven primary healthcare clinics (PHCs) in Harare, Zimbabwe.

**Methods**: Participants were recruited between January 2013 and December 2014 and followed for 18 months. Rates of and reasons for mortality, hospitalization and unscheduled PHC attendances were ascertained. Cox proportional modelling was used to determine the hazard of death, unscheduled attendances and hospitalization.

**Results**: We recruited 385 participants, median age 11 years (IQR: 9–13) and 52% were female. The median CD4 count was 375 cells/mm^3^ (IQR: 215–599) and 77% commenced ART over the study period, with 64% of those who had viral load measured achieving an HIV viral load <400 copies/ml. At 18 months, 4% of those who started ART vs. 24% of those who remained ART‐naïve were lost‐to‐follow‐up (*p* < 0.001). Hospitalization and mortality rates were low (8.14/100 person‐years (pyrs) and 2.86/100 pyrs, respectively). There was a high rate of unscheduled PHC attendances (34.94/100 pyrs), but only 7% resulted in hospitalization. Respiratory disease was the major cause of hospitalization, unscheduled attendances and death. CD4 count <350cells/mm^3^ was a risk factor for hospitalization (aHR 3.6 (95%CI 1.6–8.2)).

**Conclusions**: Despite only 64% of participants achieving virological suppression, clinical outcomes were good and high rates of retention in care were observed. This demonstrates that in an era moving towards differentiated care in addition to implementation of universal treatment, decentralized HIV care for children is achievable. Interventions to improve adherence in this age‐group are urgently needed.

## Introduction

By 2015, an estimated 1.8 million children under 15 years of age were living with HIV globally, the majority in Sub‐Saharan Africa, yet just half were accessing antiretroviral therapy (ART) [1]. While the number accessing HIV treatment represents gains from 2010, children lag disproportionately behind adults in terms of ART coverage (49% of all children infected were accessing ART in 2015 as compared to just 21% in 2010) [1].

As ART programmes have scaled up, the major barriers to ART access have been the lack of healthcare professionals to provide HIV care, overcrowding of clinics and distance to facilities where such care is available [2–6]. Among adults, the increase in numbers of individuals accessing ART has led to decentralization of HIV care provision from secondary to primary health care facilities, and task‐sharing to involve nurses in treatment of HIV infection [7,8]. The aims of decentralization and task‐sharing was to improve access to care for patients and relieve pressure on heavily overburdened secondary care facilities [9]. More recently, differentiated care initiatives are focusing on providing services which are more client focused and tailored to specific needs of diverse populations of people living with HIV [10]. Zimbabwe has incorporated the concept of differentiated care into its recent HIV treatment framework [11].

Clinical outcomes among adults HIV care in decentralized programmes with nurse‐led care have been comparable to those in secondary healthcare facilities [7,8,12,13]. Provision of HIV care to children and adolescents is associated with additional complexities, which may impact on clinical outcomes [9]. These include reliance on guardians, who are often not biological parents, for access to and retention in care, weight‐based ART dosing, difficulties in discussing HIV and disclosing HIV status to children [14–16]. Older children and adolescents with HIV have higher rates of virological failure and attrition than adults [17,18]. These have led to a relative reluctance by primary care level providers to provide HIV care to this age‐group [19]. WHO guidelines now recommend “treat all” living with HIV and Zimbabwe, since 2016, has adapted its national guidelines to reflect this. Limited data are available on ART care provision to children by nurses in primary health care facilities [20]. We present clinical outcomes among children and adolescents accessing decentralized HIV care services provided by nurses in Harare, Zimbabwe.

## Methods

### Study setting

A prospective cohort study of children aged 6–15 years was conducted in seven primary healthcare clinics (PHCs) in south west Harare, Zimbabwe between January 2013 and December 2014. Opt‐out HIV testing and decentralized, nurse‐led HIV care for children was introduced at the seven PHCs with supervision from by a physician. Children found to be HIV positive were offered enrolment into the cohort which was then followed over 18 months’ duration. Clinics where the study was performed were treating adults with HIV infection on a nurse‐led basis as per Zimbabwe national guidelines but had not been treating children living with HIV. Children were followed up by research nurses for 18 months from time of study enrolment. On completion of study follow up, HIV care and management was transferred to clinic nursing staff. Nurses in the employ of the clinic were trained on HIV care and management for children over the duration of the study. Although treatment and care was provided by research nurses with a physician backup, national guidelines for treatment and management of children living with HIV were used. Staff within the primary health care clinics where our study was performed, who similar to the model used in the study, have physician support weekly and were trained on management of paediatric HIV treatment in tandem with research nurses. Details of the study which includes baseline clinical data at time of enrolment have been described elsewhere [21]. Research nurses based at each clinic were trained on paediatric HIV testing and counselling and provision of HIV care, treatment monitoring and management of infections, based on the Integrated Management of Childhood Illness (IMCI) algorithm, over a 2‐week period prior to study commencement [22]. The Ministry of Health and Child Care training tools were utilized and simple ART‐dosing charts (available on request) were produced to facilitate weight‐based dosing. Criteria for referral to secondary level facilities were pre‐defined, including “danger signs” based on the IMCI algorithm. Nurses carried out ART eligibility screening, initiated ART and provided follow‐up care, supported by weekly visits from a physician. Adherence counselling was provided by primary care counsellors trained in paediatric HIV care. Each visit to a PHC incurs a USD1 fee which is standard throughout Zimbabwe. Cotrimoxazole and ART are provided free of charge through the National ART Programme.

### Participants

Children aged between 6 and 15 years who tested HIV positive through provider‐initiated HV testing and counselling were enrolled into the cohort study (of whom a proportion were simultaneously enrolled into a randomized controlled trial to assess impact of household support to children living with HIV[trial registry number PACTR201212000442288]), if they chose to access HIV care at the clinic where they were diagnosed and gave consent [23].

### Study procedures

At the initial assessment visit within a week of HIV diagnosis, socio‐demographic data, past clinical history and current symptoms were assessed, and a standardized examination performed. Participants underwent WHO Staging and a CD4 count, using Alere Pima™ CD4 machine, was measured [24]. All participants underwent counselling and were started on cotrimoxazole. Participants who screened positive on the WHO TB screen had sputum examined onsite by Ziehl–Neelsen smear microscopy and Xpert TB™ [25]. Participants were seen within 2 weeks of the initial visit to assess adherence and determine side effects of cotrimoxazole, and to commence ART if eligible. The schedule for follow up was based on national guidelines, with visits at 2 and 6 weeks post ART commencement and then on a 3‐monthly basis. Participants not eligible for ART at baseline underwent a 3‐monthly symptom‐based review and examination to reassess ART eligibility. At each visit, a standard proforma was used to collect information on current symptoms, side effects of ART, history of contact with primary healthcare and hospitalization since the previous visit (confirmed by patient‐held records) and history of incident infections.

Until February 2014, participants were ART eligible if CD4 was below 350 cells/mm^3^ or they had evidence of WHO Stage 3 or 4 infections. ART regimens consisted of stavudine/lamivudine and nevirapine for children under 12 years or tenofovir/lamivudine and nevirapine if over 12 years of age. Efavirenz was substituted for nevirapine in case of concomitant TB treatment or nevirapine allergy. From March 2014, Zimbabwe adopted the WHO 2013 consolidated guidelines with the threshold for ART initiation revised to 500 cells/mm^3^ and ART regimes were standardized to zidovudine/lamivudine and nevirapine for those under 12 years and not requiring TB treatment, and tenofovir/lamivudine and efavirenz for those over 12 years of age [26]. CD4 count was performed 6 monthly for all participants. HIV viral load testing was performed at 48 weeks post ART commencement using COBAS Ampliprep/Taqman 48 Version 2.0.

Unscheduled visits were defined as visits occurring outside the scheduled visits for either a medical or non‐medical reason (e.g. counselling). Side effects were graded according to the DAIDS grading system [27].Hospitalization was defined as a participant spending one night or more in a hospital. If a participant had more than one hospitalization, they were counted separately even if related to the same clinical issue. Transfer out was defined as a caregiver informing the clinic of the participant changing care to another clinic and a transfer letter being provided. Participants who failed to attend more than two scheduled appointments were traced through a phone call and/or a home visit. Participants were defined as having moved away if they had moved care to another clinic without informing clinic staff. Participants were deemed lost to follow‐up (LTFU) if they could not be traced. Tracing was performed at clinic level by research nurses who phoned the participant's guardian on 2 occasions if a participant had not returned for a visit within 3 months of the scheduled date. If after the second phone call a participant could not be reached then a voluntary lay health worker visited the house. If a participant died, the cause of death was determined through hospital records. In the case of death occurring outside of hospital, this was confirmed through verbal autopsy with the caregiver.

### Data management and analysis

Data was extracted from paper forms using optical mark recognition software (Cardiff TELEFORM Intelligent Character, Version 10.7) and analysed using STATA, version 12.1 (STATA Corporation, USA). Stunting and wasting were defined as a height‐for‐age z‐score and a weight‐for‐age z‐score of <‐2, respectively [28]. Rates of hospitalization, unscheduled visits and death were calculated. Cox proportional modelling was used to determine the hazard of death, unscheduled visits and hospitalization, controlling for factors found to be significantly associated with the outcome in univariate analysis.

### Ethical considerations

Written informed consent was obtained from all caregivers and written assent obtained from participants. Ethical approval for the study was obtained from the Medical Research Council of Zimbabwe, the Harare City Health Department Ethics Committee, the Biomedical Research and Training Institute Institutional Review Board and the London School of Hygiene and Tropical Medicine Ethics Committee.

## Results

A total of 385 participants were enrolled into the study and provided 450 person‐years of follow up and a median of 504 days (IQR 1–515). The median age at HIV diagnosis was 11 years (IQR 9–13) and 52% were female. Most participants were infected with HIV through mother‐to‐child transmission, and 59% were single or double orphans (Table 1). The median CD4 count at HIV diagnosis was 375 cells/mm^3^ (IQR 215–599). Over the 18‐month period, 296 (77%) participants commenced ART, 70% of whom did so within 4 weeks of enrolment (Table 1). Of the 89 participants who did not initiate ART, 7 were eligible according to national guidelines and 82 were not eligible during follow‐up.

**Table 1:** Baseline characteristics of enrolled participants at baseline (*n* = 385)

Characteristic	*N*
Age (years), median (IQR)	11 (9,10,11,12,13)
Female	199 (52%)
Median (IQR), CD4 cells/mm^3^	375 (215–599)
CD4< 350 cells/mm^3^	177 (46%)
CD4 350–500 cells/mm^3^	74 (19%)
CD4> 500 cells/mm^3^	131 (34%)
WHO Stage 3 or 4	
Mode of HIV acquisition	155 (40%)
Mother‐to‐child	369 (96%)
Horizontal	13 (3%)
Unknown	3 (1%)
Orphanhood	157 (41%)
Both parents alive	77 (20%)
Maternal Orphan/Father Alive	71 (18%)
Paternal Orphan/Mother Alive	58 (15%)
Double Orphan	22 (6%)
Unknown status of either parent	
Biological parent as the current caregiver	220 (57%)
Started ART within 4 weeks of enrolment	206 (54%)
Started on ART over 18‐month follow up	296 (77%)
Median days on ART for those who initiated (IQR)	485 (359–495)

**Figure 1:**
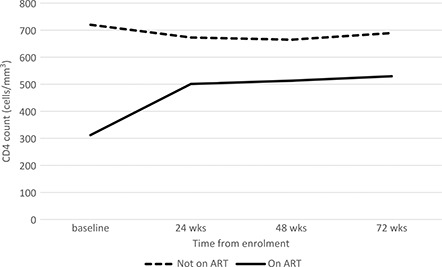
Median CD4 count over 18 month follow up by ART status.

At the end of 18 months, 286 (74%) were still in care, 50 (13%) had transferred to another clinic, 12 (3%) moved to another clinic without informing clinic staff, 9(2%) moved away and did not transfer to another clinic, 13 (3%) died, 1 withdrew from the study after initial assessment and 14 (4%) were LTFU (Table 2). Importantly, those who did not start ART were significantly more likely to be LTFU than those who started ART (*p* < 0.001). CD4 counts for those who commenced ART increased over the follow‐up period, with those who did not start ART (*n* = 89) maintaining their CD4 counts (Figure 1). Of those who commenced ART within 24 weeks of enrolment into the study (*n* = 273), 200 had a viral load sample collected between 40 and 72 weeks post ART, with 195 results obtained. 124 (64%) of those whose viral load result was obtained had a viral load <400 copies/ml.

**Table 2:** Outcomes at 18 months of cohort participants by ART initiation status

	Total *N* = 385	Initiated ART over follow up period *N* = 296	Did not initiate ART over follow up period *N* = 89	*p*‐value
In care to end of study follow up	286 (74.3%)	243 (82.1%)	43 (48.3%)	<0.01
Planned transferred to another clinic	50 (13.0%)	30 (10.1%)	20 (22.5%)	<0.01
^a^Left area without transfer of care	9 (2.3%)	2 (0.7%)	7 (7.9%)	<0.01
^a^Not in care and untraceable	14 (3.6%)	6 (2.0%)	8 (9.0%)	<0.01
No planned transfer, but was found in care at another clinic	12 (3.1%)	3 (1.0)	9 (10.1)	<0.01
Withdrew from study	1 (0.3%)	0 (0.0%)	1 (1%)	0.06
Died	13 (3.4%)	12 (4.1%)	1 (1%)	0.18

^a^ascertained through phone calls and home visits.

### Unscheduled visits

There were 146 unscheduled visits made by 99 participants over the follow‐up period, equivalent to a rate of 34.94/100 person years (95% CI 29.70–41.09). Ten unscheduled visits resulted in hospitalization, all of whom were receiving ART. The most common reason for an unscheduled attendance to the clinic was illness (n = 133, 91%). Other reasons included medication collection (n = 5, 3%) and additional adherence counselling (*n* = 8, 6%). The major cause of illness leading to an unscheduled visit was respiratory tract infection (responsible for 45% of unscheduled visits due to illness), followed by skin infections which accounted for 22% of unscheduled visits due to illness (Table 3).

**Table 3:** Reasons for unscheduled visits due to illness (*n* = 133)

Cause of unscheduled attendance	*N* (%)
Respiratory tract infection	**60 (45%)**
Upper respiratory tract infection	27
Lower respiratory tract infection	16
Pulmonary TB	11
Otitis Media	2
Tonsillitis	4
Skin infections	**29 (22%)**
Oro‐labial Herpes Simplex	5
Herpes Zoster	5
Chicken Pox	1
Bacterial skin infection	15
Fungal skin infection (*Tinea capitis* or corporis)	2
Papular pruritic eruption	1
Gastrointestinal disease	**16 (12%)**
Gastroenteritis	9
Chronic diarrhoea	2
Hepatitis A	1
Oral candidiasis	3
Oesophageal candidiasis	1
Antiretroviral therapy side effects	**20 (15%)**
Grade 1 Nevirapine hypersensitivity	8
Grade 2/3 NNRTI skin hypersensitivity	8
Grade 2/3 vomiting	3
Anaemia	1
Miscellaneous	**8 (6%)**
Minor trauma	1
Gingivitis	2
Conjunctivitis	2
Mumps	1
TB lymphadenitis	2

Side effects from ART resulted in unscheduled visits in 20 patients (15% of unscheduled visits due to illness). Of these, Grade 1 nevirapine hypersensitivity was the most common side‐effect (*n* = 8). Only one participant was hospitalized due to ART side effects‐for rehydration secondary to grade 3 vomiting. Other ART side effects reported at routine 3‐monthly follow up were uncommon and self‐limiting‐consisting of nausea (6%), vomiting (6%), abdominal pain (3%), diarrhoea (6%), fatigue (5%), rash (3%), jaundice (0.1%), dizziness (3%) vivid dreams (2%), and confusion (1%). Overall, side effects of ART resulted in drug switches in 11 cases (*n* = 9 due to nevirapine hypersensitivity, n = 1 anaemia due to zidovudine and *n* = 1 grade 3 efavirenz hypersensitivity reaction). Such switches were managed in the primary care clinics by nursing staff with consultation from study physician.

### Hospitalizations

There were 34 hospitalizations in 27 participants, 9 of which resulted in death. The rate of the hospitalization was 8.14/100 person years (95% CI 5.81–11.39). Lower respiratory tract disease was commonest reason for admission (Table 4). TB was the cause of hospitalization in 8 participants diagnosed through sputum testing using geneXpert or based on chest X‐ray findings. Six participants were hospitalized more than once for the same clinical diagnosis (1 admitted twice for HIV‐related anaemia, 1 admitted twice for lower respiratory tract infection, 1 admitted twice for Steven Johnson's syndrome secondary to cotrimoxazole due to ongoing symptoms, and 1 admitted thrice for recurrent lower respiratory tract infection). Two participants were admitted twice but for different clinical events. Only three hospitalizations occurred in participants not taking ART, two of which were due to malaria. Only one was HIV related, namely HIV‐associated anaemia and thrombocytopenia, and resulted in death. The CD4 count at hospitalization in this participant was 1114 cells/mm^3^.

**Table 4:** Causes of hospitalization (*n* = 34)

Cause of hospitalization	*N* (%)
Respiratory Illness	*n* = 18 (53%)
Pulmonary TB	7
Disseminated TB	1
Lower respiratory tract infection	9
*Pneumocystis jiroveci* pneumonia	1
Neurological Illness	*n* = 5 (15%)
Bacterial meningitis	1
Cryptococcal meningitis	2
CNS lymphoma	1
Seizure (cause unknown)	1
Miscellaneous	*n* = 11 (32%)
Hyperglycaemia	1
Anaemia^a^	3
Congestive Cardiac Failure	1
Gastroenteritis	1
Vomiting Secondary to ART	1
Malaria^b^	2
Stevens–Johnson syndrome secondary to cotrimoxazole	2

*Numbers refer to hospitalization events rather than numbers of participants admitted, ^a^n = 1 not on ART, ^b^ n = 2 not on ART*

### Deaths

The mortality rate was 2.86/100 pyrs (95% CI 1.65–4.95). Of the 13 deaths in the cohort, 12 occurred in hospital and respiratory disease was the most common cause (Table 5). The median CD4 count at enrolment of those who died was 73 cells/mm^3^ (IQR 12–205) and 77% of those who died had WHO Stage 3 or 4 disease at enrolment. The median time from enrolment to death was 76 days (IQR 59–410).

**Table 5:** Causes of death amongst cohort participants over 18 month follow up (*n* = 13)

Cause of death *n* (%)	
Pulmonary TB	4 (31)
Lower respiratory tract infection	4 (31)
Malignancy – CNS lymphoma	1 (7)
Congestive cardiac failure	1 (7)
Meningitis	1 (7)
Accidental drowning	1 (7)
Anaemia/Thrombocytopenia	1 (7)

A CD4 count less than 350 cells/mm^3^ at enrolment, WHO stage 3 or 4 HIV disease and wasting were associated with hospitalization and death on univariate analysis (Table 6). CD4 count less than 350cells/mm^3^ and advanced WHO stage remained significantly associated with the outcome in multivariate analysis for hazard of hospitalization (aHR 3.6 (95%CI 1.6–8.2), aHR 2.6 (95% CI 1.1–6.2)), no variables remained significantly associated with the hazard of death. Being older (HR 2.1 (95%CI 1.4–3.1), WHO stage 3 or 4 disease (HR 1.5 (95% CI 1.0–2.1) and wasting (HR 1.8 (95% CI 1.3–2.7)) were associated with having unscheduled visits due to illness. On multivariate analysis, association with being in an older age group (aHR 1.9 (95%CI 1.3–3.1)) and wasting (aHR 1.8(95%C.I 1.0–2.3)) remained significant.

**Table 6:** Cox proportional hazard ratio for hospitalization, unscheduled visit due to illness and death

			Hospitalization *N* = 34				Unscheduled visit *N* = 118				Death *N* = 13			
		Total	*N*	Rate (95% CI)	Crude hazard ratio	AHR^a^	*N*	Rate (95% CI)	Crude hazard ratio	AHR^a^	*N*	Rate (95% CI)	Crude hazard ratio	AHR^a^
Age	**11–15**	200	17	7.7 (4.8–12.5)	0.9 (0.5–1.8)	‐	82	37.3 (30.1–46.4)	2.1 (1.4–3.1)	1.9 (1.3–2.9)	7	3.0 (1.4–6.2)	1.1 (0.4–3.3)	‐
	**6–10**	185	17	8.6 (5.3–13.8)	1		36	18.2 (13.1–25.2)	1		6	2.8 (1.2–6.1)	1	
Sex	**Female**	199	15	6.9 (4.2–11.5)	0.7 (0.4–1.4)	‐	68	31.4 (24.7–39.8)	1.3 (0.9–1.8)	‐	9	3.8 (2.0–7.4)	1.6 (0.5–5.5)	‐
	**Male**	186	19	9.44 (6.0–14.8)	1		50	24.9 (18.8–32.8)	1		4	1.8 (0.68–4.9)	1	
CD4 at enrolment ^b^	**<350**	177	26	13.1 (8.9–19.3)	4.1 (1.8–9.4)	3.6 (1.6–8.3)	65	32.8 (25.7–41.8)	1.4 (0.9–1.9)	‐	11	5.2 (2.9–9.3)	5.0 (1.1–23.1)	4.2 (0.9–19.8)
	**>350**	205	7	3.2 (1.5–6.8)	1		53	24.5 (18.7–32.0)	1		2	0.8 (0.2–3.4)	1	
WHO stage	**3–4**	155	25	14.8 (10.0–21.9)	4.0 (1.9–8.6)	2.6 (1.1–6.2)	59	34.9 (27.1–45.1)	1.5 (1.0–2.1)	1.2 (0.8–1.9)	10	5.4 (2.9–10.0)	4.1 (1.1–15.2)	2.5 (0.6–10.7)
	**1–2**	230	9	3.6 (1.9–6.9)	1		59	23.7 (18.4–30.6)	1		3	1.1 (0.3–3.5)	1	
ART within 4 weeks	**Y**	206	22	9.2 (6.1–14.0)	1.4 (0.7–2.9)	‐	69	28.9 (22.8–36.5)	1.1 (0.75–1.6)	‐	10	3.9 (2.1–7.3)	2.1 (0.6–7.9)	‐
	**N**	179	12	6.7 (3.8–11.8)	1		49	27.3 (20.0–36.6)	1		3	1.5 (0.5–4.7)	1	
Stunting	**Y**	91	13	13.0 (7.5–22.4)	1.9 (1.0–3.8)	‐	31	31.0 (21.8–44.1)	1.1 (0.74–1.7)	‐	4	3.7 (1.4–9.9)	1.8 (0.45–6.0)	‐
	**N**	294	21	6.6 (4.3–10.1)	1		87	27.4 (22.2–33.8)	1		9	2.6 (1.4–5.0)	1	
Wasting	**Y**	105	19	17.3 (11.1–27.2)	3.5 (1.8–6.8)	2.1 (1.0–4.5)	47	42.9 (32.2–57.1)	1.8 (1.3–2.7)	1.5 (1.0–2.3)	7	5.9 (2.8–12.4)	4.7 (1.4–16.2)	2.7 (0.7–10.6)
	**N**	280	15	4.9 (2.9–8.1)	1		71	23.0 (18.3–29.1)			6	1.8 (0.8–4.0)	1	

^a^Adjusted for factors significantly associated with the outcome in univariate analysis; AHR: adjusted hazard ratio; ^b^ data missing for *n* = 3

## Discussion

Our study demonstrated that nurse‐led HIV care for children and adolescents is possible in primary care settings. The rate of retention in care was comparable to that reported in facility‐based settings and higher than a recent retrospective cohort review of children attending decentralized care in Swaziland [20,29]. Provision of HIV care in primary care facilities reduces the distance that patients need to travel to access treatment, and this may help improve retention in care [3,20]. Notably, the rate of retention in care was significantly lower in those who did not start ART, with half of those who did not initiate ART being LTFU. This finding has been reported previously and may be because patients perceive no benefit in attending for care if they are not receiving treatment [30,31]. The 2015 WHO guidelines recommend initiation of ART regardless of disease stage or age and this may facilitate retention in care [32].

Worryingly, only two‐thirds of children achieved virological suppression after starting ART, similar to the proportion reported in a recent study in a clinic in a central hospital in Harare [33]. Other studies have demonstrated virological suppression rates ranging from 27 to 89% in adolescents [34]. Notably, 172 (44.7%) of our study cohort received community lay worker support due to participation in a randomized controlled trial which may have potentially increased the proportion of participants with viral load suppression. There were no drug‐stock outs over the duration of our study and so the virological unsuppressed rate most likely reflects suboptimal adherence. Since study completion, viral load testing has become available at primary care clinics within Zimbabwe's health service. Children are expected to take ART for at least two decades longer than adults and the need for interventions in this age‐group to support sustained adherence to minimize emergence of drug resistance cannot be over‐emphasized, particularly in resource‐limited settings where options for second and third‐line ART are limited.

Despite a good immunological response to treatment, a high frequency of PHC attendances occurred outside scheduled clinic appointments. Most unscheduled presentations were, however, due to minor illness and were managed at primary care level, with only a minority resulting in referral to hospital for further management. Such referrals were decided on due to the nature of the limited services primary care health facilities are able to provide in the event of more serious illness. Decentralized HIV care therefore provides a system for triage and reduces the burden on secondary health facilities, making it a sustainable system for chronic care provision.

The leading cause of unscheduled visits, hospitalization and death was respiratory tract infections, even among patients on ART. We have previously reported a high burden of chronic lung disease in perinatally infected children and adolescents, which is associated with considerable morbidity including recurrent respiratory tract infections, poor lung function and reduced exercise tolerance [35]. The recurrent respiratory tract infections observed in this study may be due to underlying chronic lung disease. The pathogenesis is poorly understood but is thought to be a sequela of chronic infections and/or HIV‐mediated chronic inflammation. Once established chronic lung disease appears to be poorly responsive to ART, and children with recurrent respiratory tract infections may require further interventions such as additional prophylactic antibiotics or anti‐inflammatory agents [36].

Notably, 8 hospitalizations were TB related, 4 of them resulting in death, despite active case finding at baseline through sputum testing and at follow up through WHO symptom screening. This demonstrates the low sensitivity of the WHO TB screening tool, and reflects the paucibacillary nature of TB in children. TB preventative therapy using isoniazid prophylaxis had not been widely implemented in Zimbabwe at commencement of the study and no participant received it over the study period. Skin disease was the second most common cause of unscheduled visits to PHCs. Recurrent skin infections are strongly associated with HIV infection in children, and in high HIV prevalence settings should prompt HIV testing [37,38]. Side effects of ART accounted for only 15% of unscheduled attendances. Importantly, most patients with side effects were managed in primary care necessitating few drug switches, all effected at primary care level.

As has been reported in other studies, advanced disease stage and immunosuppression were risk factors for both hospitalization and death [39,40]. The median CD4 count at diagnosis was 375 cells/mm^3^, and the median age at diagnosis in a cohort where nearly all participants were infected perinatally was 11 years, implying an average delay of a decade in diagnosing HIV infection. Given the high HIV‐associated mortality observed in infants, there is limited awareness that a third of HIV‐infected infants survive to adolescence even without treatment [41]. Therefore, HIV testing is only offered when children present with conditions indicative of HIV infection, by which time they have often developed advanced disease [19,29]. However, at least a quarter of children retained high CD4 counts (>500 cells/mm^3^) and remained ineligible for ART based on current national guidelines. While a sub‐group of these may be true long‐term non‐progressors or elite controllers, many remain pauci‐symptomatic and may not prompt healthcare workers to offer HIV testing [33,34].

Older age was associated with more frequent unscheduled visits but not death and hospitalization. This might reflect a combination of survival bias and longer life‐time exposure of uncontrolled viremia. While these children survived into adolescence without major illness, the uncontrolled viremia may have resulted in a higher prevalence and more prominent chronic disease phenotype such as chronic lung disease.

The strengths of the study were the prospective design and that participants were actively followed up to ascertain outcomes. Detailed clinical data were collected to establish the reasons for visits to health facilities and cause of death. The study was conducted in public sector services and therefore the findings are broadly generalizable. Limitations include the lack of viral load data in all participants (73% of those eligible had viral load measured). There is a risk that the Cox proportional hazard models for death and admission may be over‐fitted due to a small number of events. To mitigate this risk, no variables were specified a priori. Diagnoses such as respiratory tract infections were made in PHC were based on symptoms and clinical examination, as diagnostic facilities are limited.

## Conclusions

Our study shows that decentralized nurse‐led HIV care for children is possible and results in clinical outcomes comparable to those reported in children elsewhere in southern Africa [20]. Implementation of 2015 WHO guidelines that recommend universal treatment of all HIV‐infected individuals, is likely to result in a substantial increase in children eligible for treatment, particularly given the current low ART coverage. Considerable investment in age‐appropriate HIV testing strategies, training and support for primary health care providers and interventions to support adherence need to be strengthened to achieve universal access and optimum treatment outcomes among children and adolescents.

## Competing Interests

The authors have no conflict of interests

## Authors’ contributions

RAF designed the study. GMCH and ED supervised data collection. GMCH and VS analysed the data. GMCH wrote the first draft of the manuscript. All authors contributed to the writing of the manuscript.

## Acknowledgements

We wish to acknowledge the study participants, their caregivers and our dedicated research nurses and assistants.

## Funding

The study was funded by the Wellcome Trust though an Intermediate Fellowship Grant (095875/Z/11Z) awarded to RAF. The authors have no conflict of interests.
